# Supplementary figures and images for: Discovery and Genomic Analysis of Three Novel Viruses in the Order Mononegavirales in Leafhoppers
Source: Viruses. 2024 Aug 19;16(8):1321. doi: 10.3390/v16081321 (PMC11360795; doi:10.3390/v16081321)

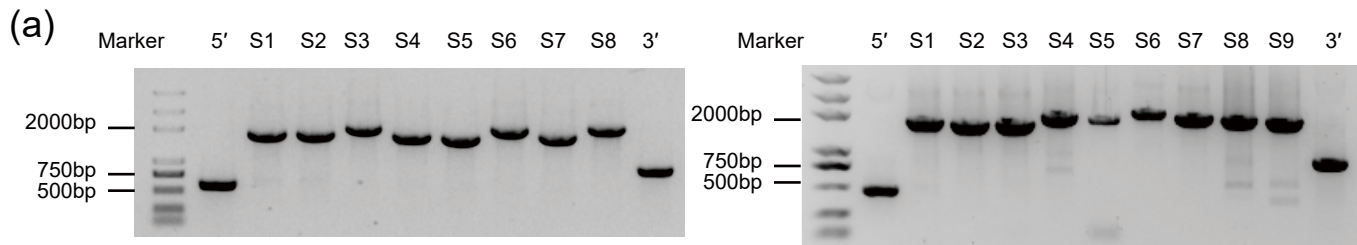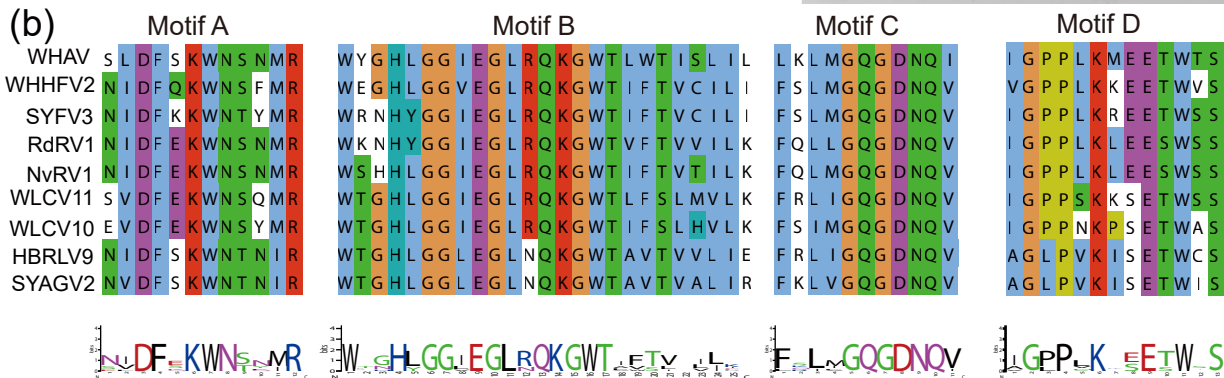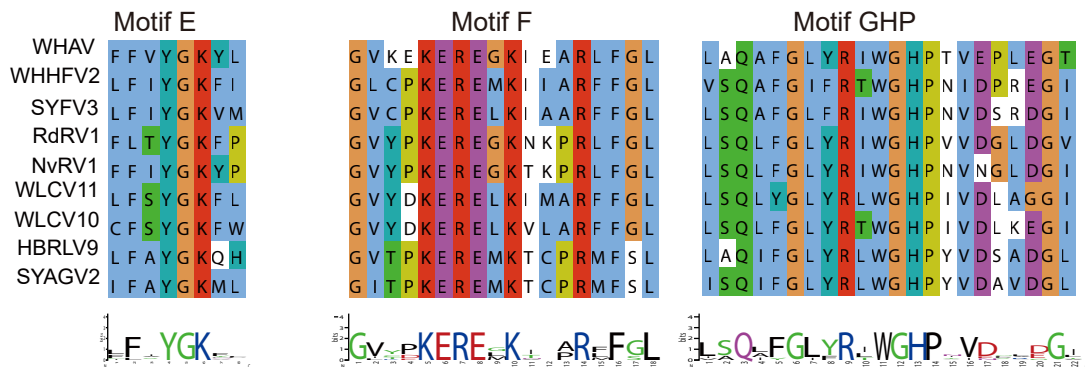

Supplement: Supplementary file 1 [file viruses-16-01321-s001.zip › Figure S1.pdf]

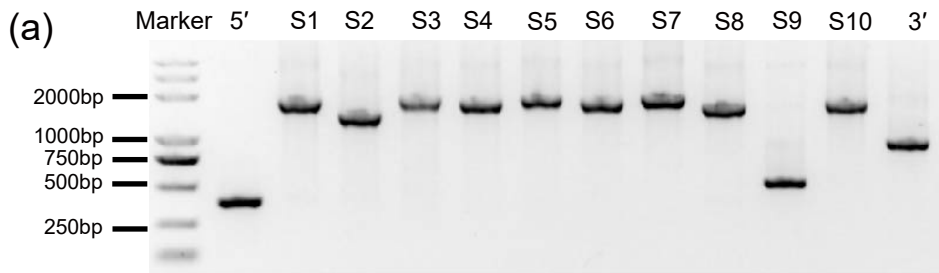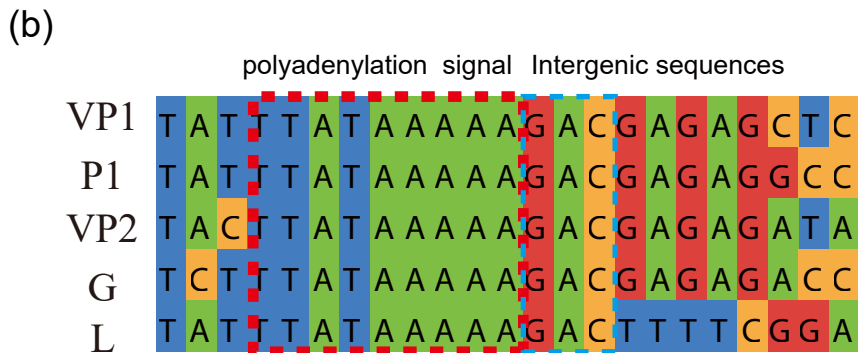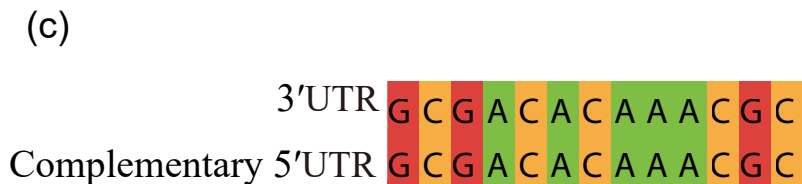

Supplement: Supplementary file 1 [file viruses-16-01321-s001.zip › Figure S2.pdf]
